# Supplementary material for: Engineering solutions for biological studies of flow-exposed endothelial cells on orbital shakers
Source: PLoS One. 2022 Jan 21;17(1):e0262044. doi: 10.1371/journal.pone.0262044 (PMC8782315; doi:10.1371/journal.pone.0262044)
Supplement: S1 File — (PDF) [file pone.0262044.s001.pdf]

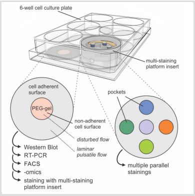

Engineering solutions for biological studies of flow-exposed endothelial cells on orbital shakers

Andreia Fernandes[1], Vahid Hosseini[1], Viola Vogel[1], Robert Lovchik[2]

[1]Eidgenössische Technische Hochschule Zürich; [2]IBM Research Europe

dx.doi.org/10.17504/protocols.io.b2bwqape

Robert Lovchik

Shear stress is extremely important for endothelial cell (EC) function. The popularity of 6-well plates on orbital shakers to impose shear stress on ECs has increased among biologists due to their low cost and simplicity. One characteristic of such a platform is the heterogeneous flow profile within a well. While cells in the periphery are exposed to a laminar and high-velocity pulsatile flow that mimics physiological conditions, the flow in the center is disturbed and imposes low shear stress on the cells, which is characteristic of atheroprone regions. For studies where such heterogeneity is not desired, we present a simple cell-patterning technique to selectively prevent cell growth in the center of the well and facilitate the exclusive collection and analysis of cells in the periphery. This guarantees that cell phenotypes will not be influenced by secreted factors from cells exposed to other shear profiles nor that interesting results may be obscured by mixing cells from different regions. We also present a multi-staining platform that compartmentalizes each well into 5 smaller independent regions: four at the periphery and one in the center. This is ideal for studies that aim to grow cells on the whole well surface, for comparison with previous work and minimal interference in the cell culture, but require screening of markers by immunostaining afterwards. It allows to compare different regions of the well, reduces antibody-related costs, and allows the exploration of multiple markers essential for high-content screening of cell response. By increasing the versatility of the 6-well plate on an orbital shaker system, we hope that these two solutions motivate biologists to pursue studies on EC mechanobiology and beyond.

DOI

dx.doi.org/10.17504/protocols.io.b2bwqape

Andreia Fernandes, Vahid Hosseini, Viola Vogel, Robert Lovchik . Engineering solutions for biological studies of flow-exposed endothelial cells on orbital shakers. **protocols.io**
https://dx.doi.org/10.17504/protocols.io.b2bwqape

orbital shaker, shear stress, cell culture, mechanobiology

protocol ,

Nov 24, 2021

Dec 01, 2021

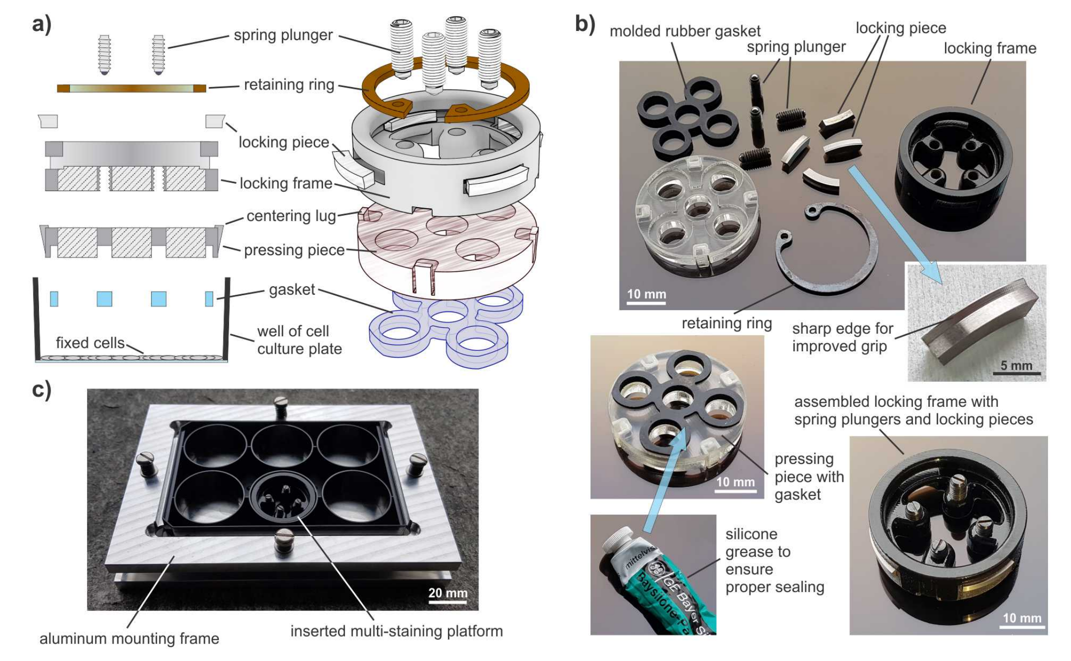

Figure 3 – Parts of the multi-staining platform. a) Overview schematics depicting the different pieces of the staining platform and assembly order. **b)** Photographs of the different pieces and materials used for the assembly of a multi-staining platform insert. **c)** Photograph of the aluminum mounting frame, containing a glass-bottom 6-well plate with an inserted multi-staining platform.

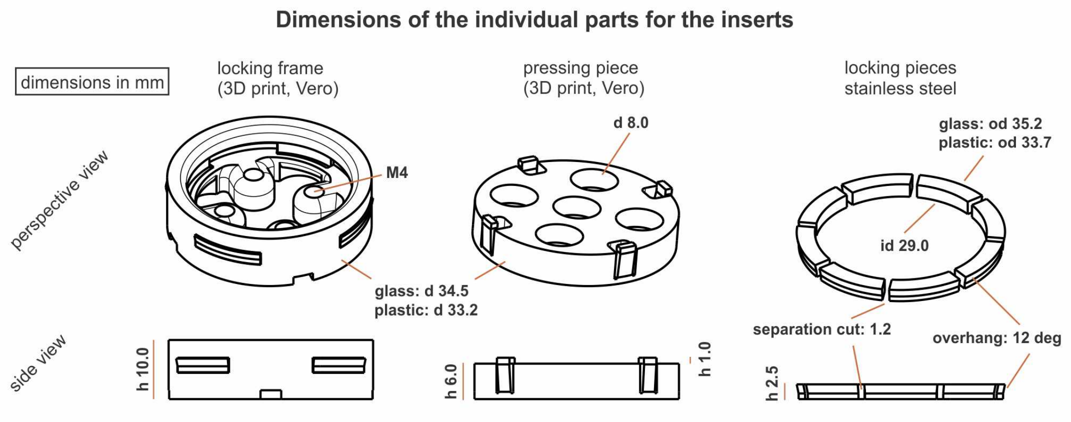

Figure 4 – Dimensions and material of custom-made parts. The locking frame and pressing piece are 3D printed and the locking pieces conventionally manufactured from stainless steel.

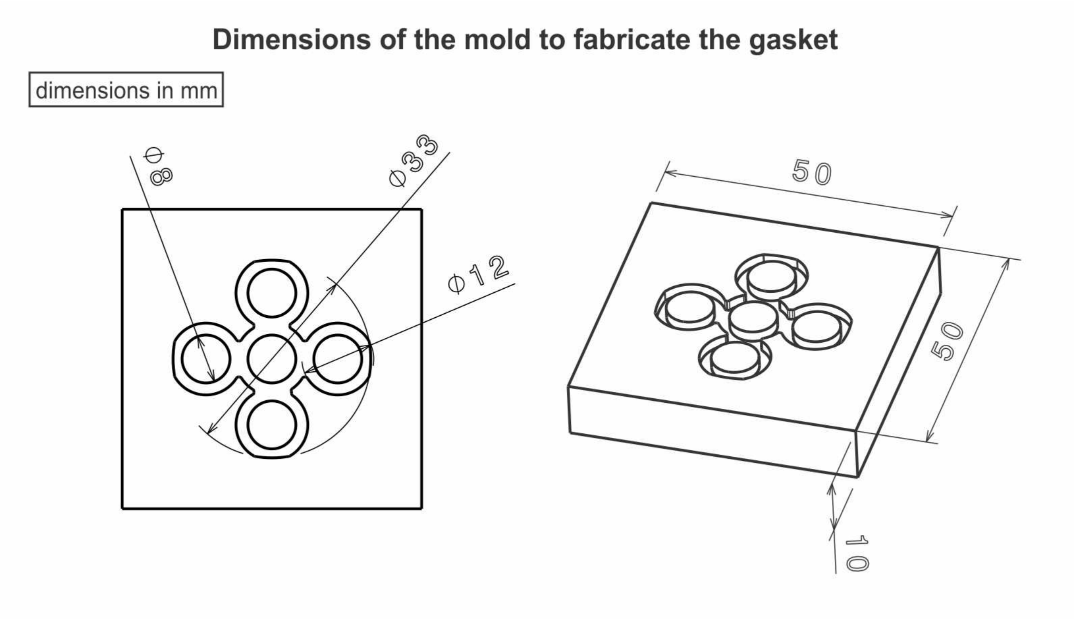

Figure 5 –Technical drawing of the mold for the gasket fabrication. The mold is fabricated using CNC machining and the material is preferably Teflon® or another “inert” material (e.g. POM). The depth of the mold is 2 mm. A soft polymer resin (e.g. PDMS or Flexane) is poured into the milled groove and then covered with a glass slide for curing. The glass slide needs to be coated with a non-sticking reagent, such as a fluorinated silane or other standard coatings for silicone molding.

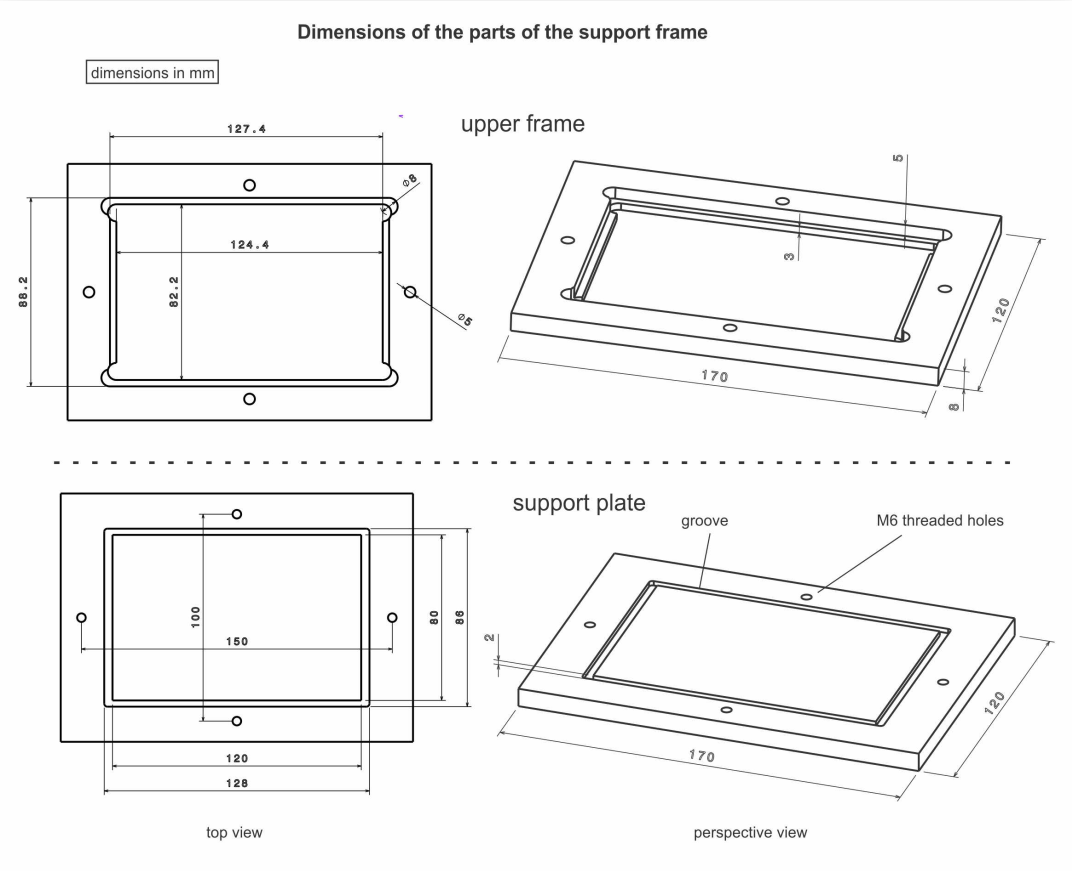

Figure 6 – Dimensions of the support frame for the multi-well plate. This frame is designed to support the glass-bottom multi-well plates, so that the pressure applied by the multi-staining inserts does not damage the glass. The pieces for the support frame were conventionally manufactured from aluminum.

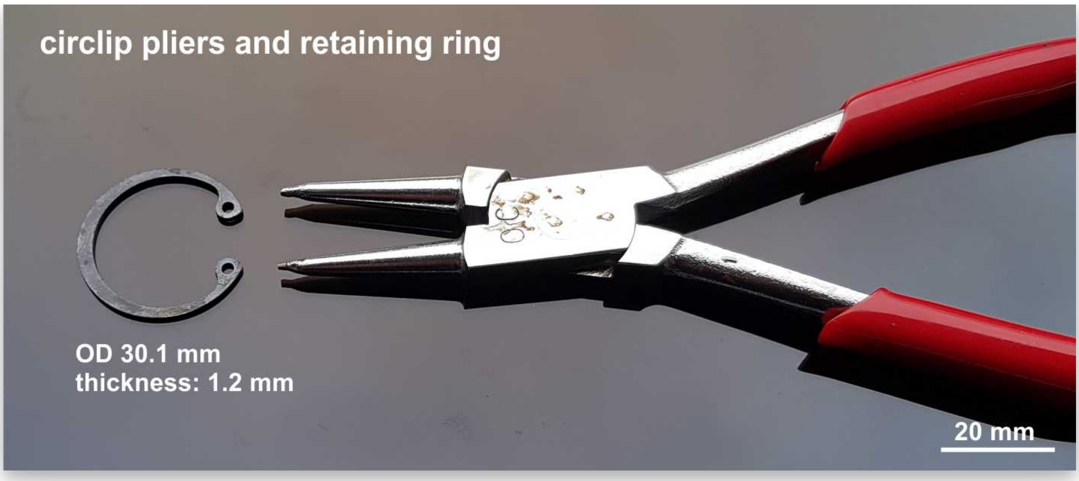

Figure 7 – Photograph of circlip pliers and retaining ring. For both versions of inserts (plastic-bottom or glass-bottom plates) retaining rings with OD 30.1 and a thickness of 1.2 mm were used (left). The retaining rings are standard parts and commercially available in many sizes. The circlip pliers (right) are special pliers with curved-in edges used to compress retaining rings.

Cell culture

- **Human umbilical endothelial cells (HUVECs)** were cultured up to passage 6 in Endothelial Growth Medium (EGM-2) supplemented with EGM™-2 BulletKit™(Lonza). Singe donor primary cells isolated by collaborators at the University Hospital were used.
- **Human Aortic Smooth Muscle Cells (HASMCs),** from Lonza, were cultured up to passage 7 in Smooth Muscle Cell Growth Medium (SmGM, Promocell).

CAD data

- **Mold_for_PDMS_gasket.stp**
- **Titerplate with support plate.stp**
- **clamping pieces.stp**
- **Assembly for plastic bottom plates.stp**
- **Assembly for glass bottom plates.stp**

PROTOCOL 1:

1 Coating the center of cell culture wells with non-adherent PEG gel

1.1 Draw a cross to locate the center of the well and mark the limits of a virtual circle around the center with 4 points at a 6 mm distance from the edge of the well. For glass-bottom plates, place a sterile filtered 10 µL drop of 3-(trimethoxysilyl) propyl methacrylate (TMSPMA) 1% v/v in methanol in the center of the well.

1.2 Using a pair of tweezers, place a sterilized 12 mm diameter coverslip on top of the droplet.

1.3 Keep the plate at room temperature for 30 minutes and subsequently incubate in the oven at 80 °C for 30 minutes.

1.4 After cooling down, fill the well with phosphate-buffered saline (PBS) and remove the coverslip. For plastic dishes, the TMSPMA treatment (steps 1-3) can be skipped.

1.5 Add 10 µL of PEG Mw3400 10% w/v + 0.5% w/v Irgacure 2959 (Material 55047962), dissolved in PBS, to the center and cover with a sterile 12 mm diameter coverslip.

1.6 After exposing the well to UV light for 30 seconds, remove the coverslip under PBS using a pair of tweezers.

1.7 Remove PBS and let the well dry.

1.8 Incubate the well with 0.2% gelatin solution for 40 minutes at 37 °C.

1.9 Wash with PBS.

1.10 Sterilize with UV light for 15 minutes.

1.11 The well is now ready for cell culturing on a shaker.

2

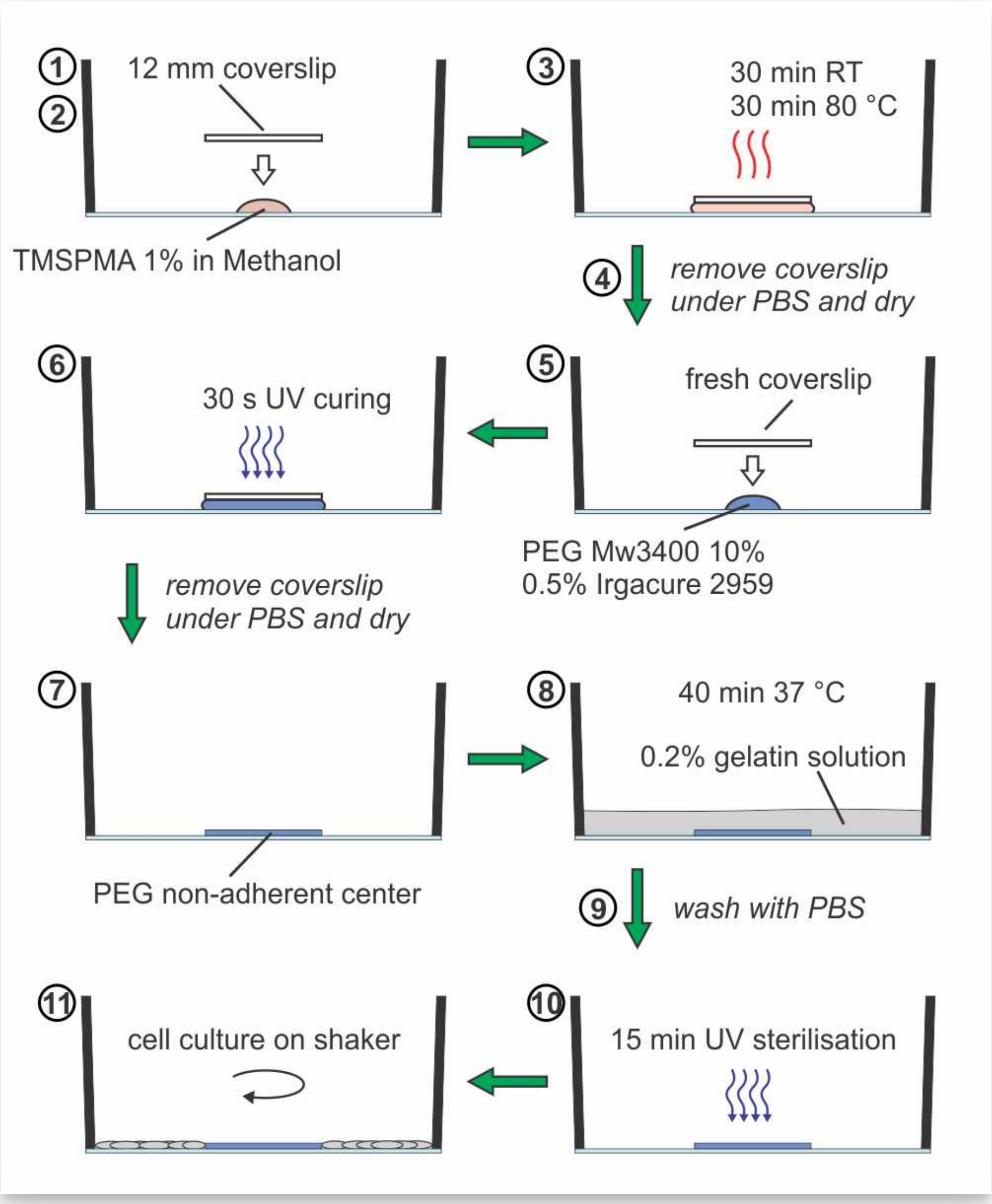

Figure 2 – Step-by-step description of the method to block cell adhesion in the center of the well using a PEG-hydrogel. (1-6) Placing a drop of TMSPMA at the center of the well. Covering the droplet with a glass coverslip for incubation. Incubation with PEG Mw3400 and Irgacure 2959 using a fresh coverslip and curing through UV exposure.(8, 9) Coating of the well with a 0.2% gelatin solution and washing. (10) Sterilization with UV light. (11) Cell culturing on an orbital shaker.

Remark:

- To harvest protein or RNA, a cell scraper can be used, and one should be careful not to collect the PEG-coating as well.

PROTOCOL 2:

3 Fabrication and assembly of the multi-staining platform

The first part of this protocol explains how to fabricate the multi-staining inserts and the supporting frame that prevents the glass bottom wells from breaking. The second part describes the assembly steps of the platform inserts into a well of a 6-well plate.

4 Fabrication steps for the multi-staining platform:

Figures 3 to 7 (see Materials section) provide illustrations for better understanding. Figure 8 shows an example of the provided CAD drawings of the different parts.

4.1 The locking frame and the pressing piece (Figure 3a-b and 4) can be 3D printed using the STEP (*.stp) files provided (we used a Stratasys Eden 260VS printer with the resins Vero Clear or Vero Black). The centering lugs (Figure 3a-b and 4) are designed to avoid any side movement of the pressing piece. They can deflect about 0.25 mm inwards when the pressing piece is installed. The lower radius of the pressing piece is about 0.1 mm smaller than the lower radius of the wells to provide some freedom of movement.

4.2 The rubber gaskets can be molded using machined Teflon® templates (Figure 5).

a. For the study, two different materials were used to fabricate the gaskets, PDMS (Sylgard® 184, Dow Corning) and Flexane (Devcon® Flexane 60L).
b. For both types of gaskets, pour the liquid resin into the Teflon template and ensure that no air bubbles are trapped.
c. Place glass slides (50 mm by 50 mm, 1 mm thick), which are pre-treated with 1H,1H,2H,2H-perfluorodecyl-trichlorosilane (ABCR GmbH, Karlsruhe, Germany) to avoid sticking, onto the filled templates and clamp with paper clips for curing.
d. Cure over night at 60°C for both materials.
e. Demolding of the gaskets is facilitated by using a few drops of ethanol while manually pulling the gasket out of the mold using a pair of tweezers.

4.3 The spring plungers (M4 × 9 mm, stainless steel) and retaining ring (OD: 30.1 mm, thickness: 1.2 mm) are available from standard hardware suppliers (Figure 3 a-b).

4.4 The locking pieces are preferably manufactured from stainless steel (Figure 3 a-b and 4). This involves the fabrication of a 2.5 mm thick ring with ID: 29 mm and OD: 35.15 mm, from which some additional material (approximately 0.3 mm) is removed on the outside to sharpen one edge for better clamping. The ring is then cut into 8 equally sized pieces using a milling machine equipped with a 1 mm side milling cutter.

4.5

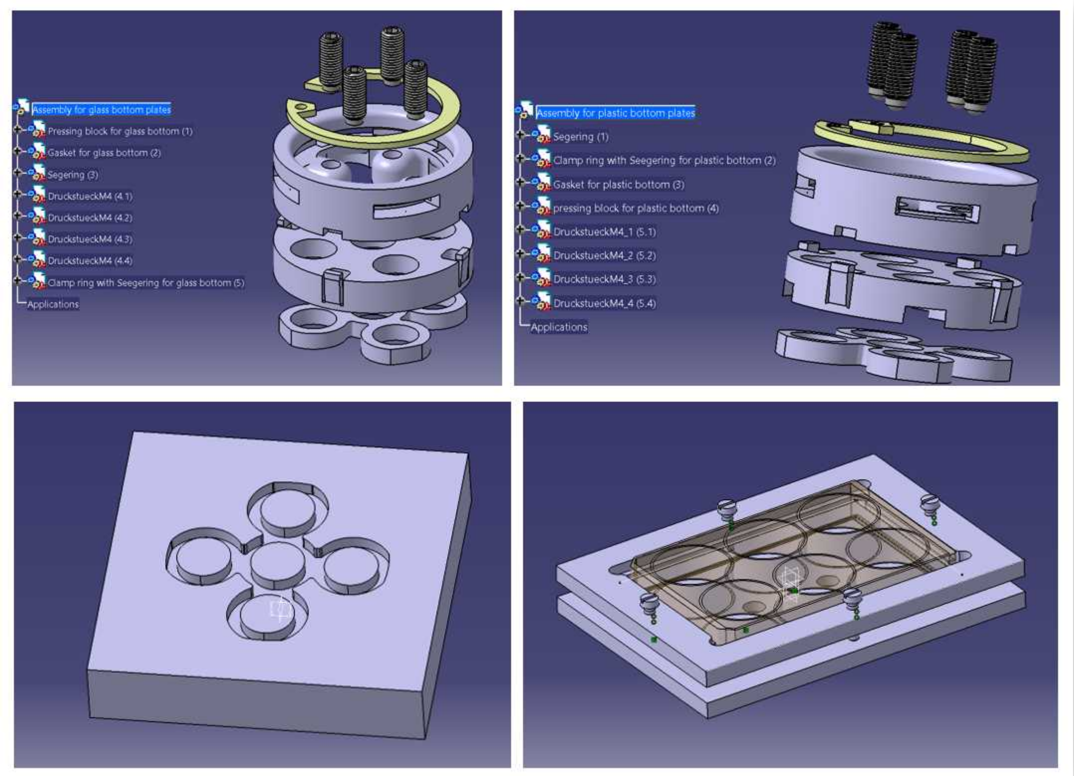

Figure 8 – Screenshots of detailed CAD drawings provided in standard STEP format. CAD drawings of both versions of inserts as well of the gasket mold and the support frame are provided. The files are in the standard CAD exchange format STEP (.stp). This format is supported by all common CAD programs and can be used for 3D printing and conventional as well as CNC machining.

Remark:

- The dimensions of the pieces presented in the study are designed to fit a glass-bottom 6-well plate from Cellvis (P06-1.5H-N), which allows for high resolution imaging. The wells in standard plastic-bottom 6-well plates have a different diameter, and the dimensions of the multi-staining platform inserts thus vary slightly in size, as described in Figure 4. The diameter of the pockets is 8 mm in both versions, resulting in 50.24 mm^2 staining area per pocket.

5 Assembly steps of the platform inserts into a well of a 6-well plate:

Figure 9 provides an illustration of the assembly steps for better understanding.

5.1 To avoid damaging of the glass at the bottom of the 6-well plates when applying a vertical force, secure the plate into the mounting frame. For plastic-bottom plates, this step can be ignored.

5.2 Insert the rubber gasket into the retainer of the pressing piece. This gasket is essential for sealing the individual pockets and avoiding fluidic cross-contamination between the compartments. For better sealing, a thin layer of hydrophobic silicone paste can be applied to the lower gasket surface.

5.3 Remove any liquid present in the well before the pressing piece with the mounted

gasket is carefully inserted into the well and placed directly on top of the fixed cells. It is important to avoid lateral movements while inserting the pressing piece to prevent damaging the biological sample.

5.4 Insert the four locking pieces into the retainers of the locking frame with the sharp edge facing upwards.Partially screw the spring plungers into the locking frame by hand.

5.5 Using circlip pliers (Figure 7), compress the retaining ring to fit into the groove inside the locking frame. The lateral force dislocates the locking pieces outward towards the wall of the well. This clamping mechanism locks the insert to the wall of the well and prevents any kind of movement.

5.6 Tighten the spring plungers using a screwdriver. This will apply force to the pressing piece and thereby completely seal the compartments. The spring plungers should not be completely compressed, thus when a “clicking” sound is heard, suggesting that it started to compress, only one additional half-turn of tightening should be added.

5.7 Finally, fill the individual compartments with staining reagents (about 100 µL each). Since drought of the tissues can compromise the quality of immunostainings, this entire procedure needs to be performed quickly, preferentially within less than one minute. We advise that the parts be pre-assembled and ready to use before removing the liquid from the wells. We also recommend using PBS to test whether there is any leakage before adding the staining reagents.

5.8

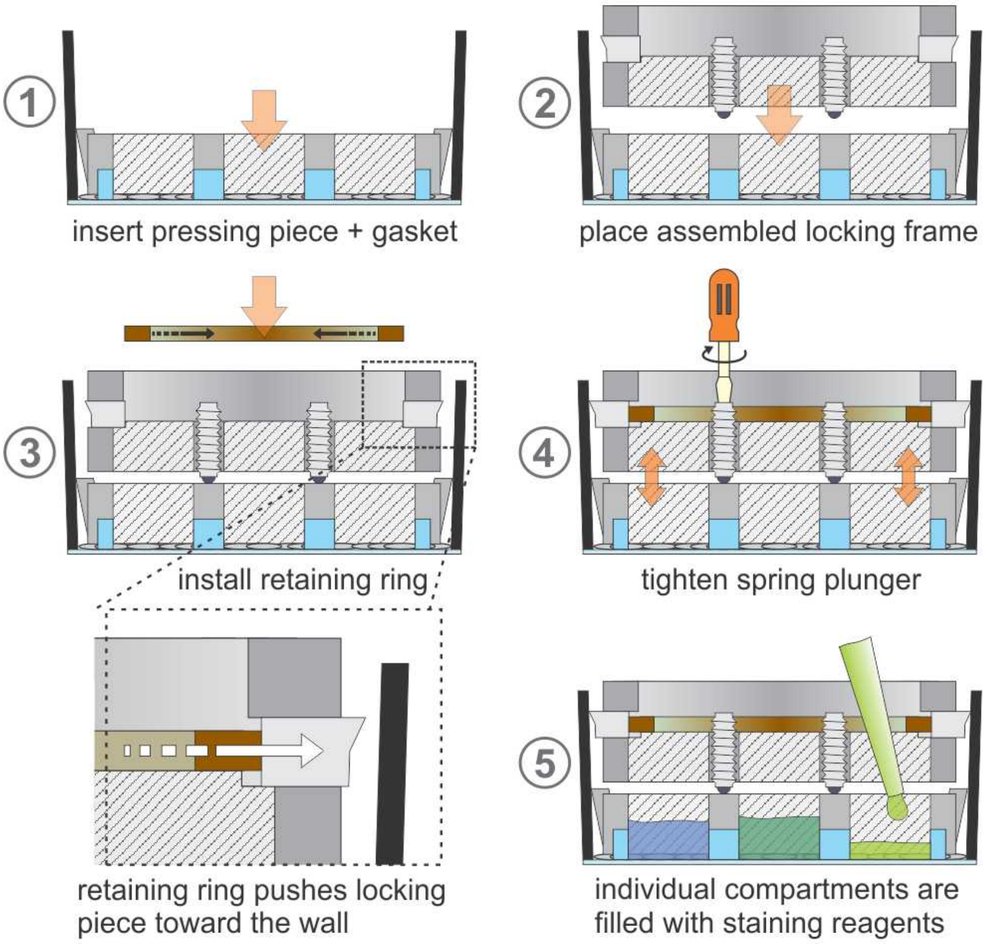

Figure 9 -Assembly of a multi-staining platform insert. Steps: (1) The pressing piece with the gasket is inserted into the well and placed directly on top of the fixed cells. (2) The locking frame is placed on top, and the centering lugs ensure proper alignment. The four locking pieces are previously inserted into the locking frame with the sharp edge facing upwards and the spring plungers inserted into the holes of the locking frame. (3) Using circlip pliers, the retaining ring is compressed to fit into the groove of the locking frame, pressing the locking pieces out towards the wall of the well. (4) The spring plungers are tightened to apply vertical force. (5) The individual compartments are filled with staining reagents.

Remarks:

- The insert can be placed inside a well after the fixation of the cells or the blocking step. To confirm the successful isolation of each staining compartment, we recommend checking the change in volume of the added liquid after a minimum of 15 minutes.
- For cell culture imaging, only the pressing piece with the gasket should remain in the well. With the removal of the locking frame the sealing can fail, and more liquid should be added to avoid tissue drought. It is crucial to keep the pressing piece in place because its removal could cause the compressed tissue to detach, and floating fragments may interfere with the imaging.
- o store a sample for a longer period, we recommend adding some PBS with 0.01% sodium azide.

PROTOCOL 3:

6 Cell culture and immunostaining

This protocol specifically relates to the cell culturing performed for the experimental part of the associated publication in PLOS-ONE (link will be added).

6.1 *For endothelial cell monocultures:*

1. HUVECs were seeded on 0.2% gelatin-coated glass 6-well plates (Cellvis, P06-1.5H-N) with a cell density of 8×10^4 cells/cm^2, using MCDB131 medium (Invitrogen) and a Low Serum Growth Supplement Kit (Invitrogen, S003K).
2. After 1-3 days of static culture, the cell layer became confluent, and the plates were placed on an orbital shaker (VWR® Advanced 3500 Orbital Shaker, radius 9.5 mm), where the cells were exposed to pulsatile laminar flow in the periphery and disturbed flow in the center. To create wall shear stress values within the physiological range, the shaker velocity was set to 135 rpm, and 2 mL of culture medium were added to each well.
3. After 3 days under flow conditions, cells had aligned with the flow and were fixed with 4% paraformaldehyde for 15 min.

6.2 *For co-cultures:*

1. HASMCs were seeded on 0.2% gelatin-coated glass 6-well plates with a cell density of 4×10^4 cells/cm^2, using SmGM.
2. After 3 days, HASMCs were stained with 10 µM CellTracker™ Green CMFDA Dye (Thermo Fisher, C7025) for 40 minutes at 37 °C.
3. After 3 long washing steps with SmGM, HUVECs were seeded at 8×10^4 cells/cm^2 on top of the HASMCs, using MCDB131 medium (Invitrogen) and a Low Serum Growth Supplement Kit (Invitrogen, S003K).
4. After 4 hours, the culture medium was exchanged and co-cultures were placed on the shaker. To create wall shear stress values within the physiological range, the shaker velocity was set to 135 rpm, and 2 mL of culture medium were added to each well.
5. After 3 days under flow conditions, the cells were fixed with 4% paraformaldehyde for 15 min.

6.3 *Immunostaining and imaging:*

1. HUVECs were permeabilized for 10 minutes with 0.1% Triton and blocked with 1% BSA for 1 hour at room temperature.
2. VE-cadherin antibody (Cell Signaling #2500, 1:500) and goat anti-rabbit antibody labelled with Alexa Fluor 488 (Invitrogen A11034, 1:250) diluted in 1% BSA were used. Incubation with primary antibodies was done overnight at 4° C and secondary antibodies for 1h at room temperature.
3. EC monoculture images were made with a Nikon Eclipse Ti2 epifluorescence microscope using Nikon NIS-Elements Advanced Research 5.02 software. EC-SMC co-culture widefield fluorescent images were created in an ImageXpress micro (IXM) from Molecular Devices using Molecular Devices MetaXpress 5 software. Images were stitched into a large mosaic using JavaScript.
