# Supplementary material for: Engineering solutions for biological studies of flow-exposed endothelial cells on orbital shakers
Source: PLoS One. 2022 Jan 21;17(1):e0262044. doi: 10.1371/journal.pone.0262044 (PMC8782315; doi:10.1371/journal.pone.0262044)
Supplement: Attachment — Submitted filename: RESPON~2.DOC [file pone.0262044.s002.DOC]

**Response to the Editor and the Reviewers**

We thank the reviewers and the editor for their comments and constructive feedback and are happy that our work is being considered for publication in PLOS ONE with minor revision.

Below we provide point-by-point replies (black font) to the specific comments of the editor and the reviewers (blue font).

**Journal Requirements:**

1. Please ensure that your manuscript meets PLOS ONE's style requirements, including those for file naming.

We adjusted the manuscript according to the PLOS ONE style requirements.

- we added line numbering,

- we adjusted the headings,

- we removed the figures from the manuscript and uploaded them separately with the correct naming and format,

- we placed the figure captions in the manuscript where they should appear, and

- we removed the supporting information from the end of the manunscript, as this is now available on Protocols.io.

2. Thank you for providing the following Protocols.io DOI in the Methods section of your manuscript [Protocols.io DOI]. In keeping with our submission requirements, please add the Protocols.io DOI in the “Protocol DOI” field of the submission form (via “Edit Submission”).

We uploaded the methods to Protocols.io in the format of a protocol and added the DOI to the Methods section in the manuscript.

The DOI for the protocol is: dx.doi.org/10.17504/protocols.io.b2bwqape

3. Thank you for stating the following financial disclosure:

a) Please clarify the sources of funding (financial or material support) for your study. List the grants or organizations that supported your study, including funding received from your institution.

This work was part of the Zurich Heart project from Hochschulmedizin Zürich and was funded by the Zurich Heart Grant and the ETHeart Initiative (ETH Zurich).

b) State what role the funders took in the study. If the funders had no role in your study, please state: “The funders had no role in study design, data collection and analysis, decision to publish, or preparation of the manuscript.”

The funders had no role in study design, data collection and analysis, decision to publish, or preparation of the manuscript.

c) If any authors received a salary from any of your funders, please state which authors and which funders.

Andreia Fernandes was funded by the Zurich Heart Grant and ETHeart Initiative (ETH Zurich).

d) If you did not receive any funding for this study, please state: “The authors received no specific funding for this work.”

N/A

Please include your amended statements within your cover letter; we will change the online submission form on your behalf.

4. Please note that in order to use the direct billing option the corresponding author must be affiliated with the chosen institute. Please either amend your manuscript to change the affiliation or corresponding author, or email us at plosone@plos.org with a request to remove this option.

N/A

5. Please amend the manuscript submission data (via Edit Submission) to include author Andreia Fernandes, Vahid Hosseini and Viola Vogel.

We are including the missing authors with the submission of the revised manuscript.

6. We note that you have included the phrase “data not shown” in your manuscript. Unfortunately, this does not meet our data sharing requirements. PLOS does not permit references to inaccessible data. We require that authors provide all relevant data within the paper, Supporting Information files, or in an acceptable, public repository. Please add a citation to support this phrase or upload the data that corresponds with these findings to a stable repository (such as Figshare or Dryad) and provide and URLs, DOIs, or accession numbers that may be used to access these data. Or, if the data are not a core part of the research being presented in your study, we ask that you remove the phrase that refers to these data.

We mentioned “data not shown” in a section where we tried to explain the difficulties encountered while developing this platform. “Failed” means that the individual pockets leaked, and therefore no data could be obtained. This troubleshooting process went on for a year and many of the used materials have been disposed in the meanwhile. It is also difficult to capture leakage in a photograph. Thus, we have removed the references to specific approaches that we tested, but we broadly mention the challenges we encountered and why such a rather complex system was necessary in the “Discussion” section.

7. Please include captions for your Supporting Information files at the end of your manuscript, and update any in-text citations to match accordingly.

Our supporting files can now be found on the Protocols.io platform.

8. Please review your reference list to ensure that it is complete and correct. If you have cited papers that have been retracted, please include the rationale for doing so in the manuscript text, or remove these references and replace them with relevant current references. Any changes to the reference list should be mentioned in the rebuttal letter that accompanies your revised manuscript. If you need to cite a retracted article, indicate the article’s retracted status in the References list and also include a citation and full reference for the retraction notice.

We have reviewed and updated the reference list. References 4, 16 and 22 are new.

General criticism:

1. The idea to avoid interference from heterogenous flow patterns (disturbed, oscillatory flow /recirculating eddies in the center vs, unidirectional, laminar flow) which elicit distinct physiological responses, is good, but not innovative. This idea has been promoted in an elegant 2018 paper by Ghim et al. The relatively small engineering innovation here is in the specific way, how the central zone of the culture dish is rendered non-adherent. A critical question, however, that has not been addressed here, is whether this really matters, i.e., whether signals originating from the cells in the disturbed central zone impact the cells elsewhere in the culture dish? The Ghim paper is quite ambivalent about this (“depends on what parameter you are looking at”). Comparisons of the physiological responses of their cells (e.g. expression / upregulation of EC activation molecules) in the laminar flow zones in the presence or the absence of cells in the center of circular culture dishes, would increase the confidence in this methodology.

In this paper we focus solely on providing technical solutions so that biological questions can be addressed, but we do not aim to address any biological questions ourselves.

We agree with the reviewer. It is still unclear whether it really matters, but the issue has been raised more than once in the literature and Ghim et al. seem to have provided the first evidence that it does. In TNF-α treated HUVEC, segmentation increased the monocyte adhesion and the expression of VCAM-1 and ICAM-1 in the center of the well but had no effect at the periphery. This indicates that cells at the periphery express mediators that reduce inflammation in the center. Knowing that ECs express very different phenotypes at the periphery compared to the center and that all secreted factors will be mixed in the culture medium, we expect that other parameters could be influenced and with this work we provide a tool for biologists to address this question.

To simply exclude cells from the center, our patterning solution is simpler than the one described by Ghim et al, since all materials are readily available in the lab. It will be especially relevant for studies where the main focus is on growing cells in the periphery or to quickly answer the question “does it matter for the parameters that are being analyzed?”.

We made sure to refer to the platform from Ghim et al. in the manuscript, for those wishing to compare the center and the periphery.

2. The proposed approach for generating a multi-staining platform is technically elegant, but, in principle, not novel: A similar idea has been published by Ghim et al., 2018. This new paper seems to build on and extend the Ghim paper. Moreover, the engineering approaches for creating this multi-staining platform, appear to be technically involved and fairly cumbersome. Previous work by others (e.g., Brooks et al., Endothelium, 11:45–57, 2004) has employed a much simpler, yet effective approach, placing small circular coverslips in various locals of a circular culture dish.

Ghim et al developed a similar, but simpler, strategy because the patterning was done on a hydrophobic surface. Because we compartmentalize a hydrophilic surface, we needed to increase the complexity. The novelty is that, to our knowledge, we are the first ones to compartmentalize a pre-existing cell layer in a 6-well plate.

Brooks et al. present a different cell patterning solution, which may be relevant for other type of studies. There are several advantages to using our staining platform compared to the one introduced by Brooks et al.:

- The platform is utilized at the end of the experiment, after cell fixation, and thus minimally influences the cell culture system (not risking contamination or affecting flow).
- Because the platform is added only at the end of the experiment, the whole well can be analyzed and the supernatants collected before compartmentalizing for the staining. This way, one does not miss possibly interesting results when looking at the “whole picture”.
- Even though the production and gathering of all necessary pieces requires some time investment at the beginning, once everything is prepared, our staining platform is assembled quickly. Each well needs to be assembled within less than 1 minute to avoid tissue drought. Coverslips are not necessarily much simpler to handle because they are fragile and require fine motor control and precision to be placed.

- Furthermore, by using our platform we can better ensure that all pockets are at the same position in the different wells, which improves comparability.
- Finally, we imagine that for staining the cells on coverslips, these may have to be removed - with the risk of breaking - or the staining on different coverslips may spill and stain neighboring coverslips in the well. These issues can be avoided by using our platform.

We also rephrased the abstract, hoping that the advantages of our proposed solutions are now more evident.

3. The authors should validate their system by computational fluid dynamics (CFD) since their experimental conditions are similar to, but no identical to those used by Ghim et al.

The fluid dynamics have been carefully described in previous publications, which are referenced throughout the manuscript (ref No. 18-20). We have used similar conditions as in Salek et al (ref 18). Note that the staining platform is only used after the experiment on the orbital shaker has been completed and cells have been fixed, thus not interfering with the flow. We clarified this further in the manuscript.

**Specific comments**

1. The authors’ description of the flow patterns in the center of the dish as “turbulent’ (3 occurrences in the text) is incorrect. Rather, it is characterized as disturbed/oscillatory. For a more quantitative, engineering approach, that authors should characterize their system by CFD.

We have corrected the flow terminology throughout the paper.

2. Immunostaining should not just be limited to visualizing VE-cadherin for EC identification, but should be extended to includee functional analysis assessing the expression/upregulation of activation molecules, such as VCAM-1 or E-Selectin.

In this paper, we focus solely on providing technical solutions so that biological questions can be addressed, but we are not trying to address any biological questions ourselves. In our work we aimed to show that high quality staining could be achieved using the platform, and that there is no leakage to other compartments.

3. Figure 3 b is not publication-quality, the distinct staining patterns (immune) are not discernible.

Additional high-quality zoom-in images have been added to the figure for better visualization of the obtained staining results.

4. Figure 4 is instructive, but in its complexity indicative for the highly complicated engineering approach. Much simpler solutions have been described in the past (see, e.g., Brooks et al., 2004)

This question has already been addressed above. Please see our response to “General criticism” Nr. 2.

5. Figure 5 , Panels a and b are not publication-quality

We improved the quality of Fig. 5a and b for publication. The images of the VE-cadherin staining and the DAPI staining are now displayed with significantly higher contrast.
